# Joseph T. Freeman Award and Excellence in Rehabilitation of Aging Persons Award Presentations and Lectures

**DOI:** 10.1093/geroni/igaf122.956

**Published:** 2025-12-31

**Authors:** Debra Bakerjian

## Abstract

The Joseph T. Freeman Award lecture will feature an address by the 2025 Freeman Award recipient Jeff Williamson, MD, FGSA and the 2025 Excellence in Rehabilitation of Aging Persons Award recipient Sajay Arthanat, PhD. The Joseph T. Freeman Award is a lectureship in geriatrics awarded to a prominent clinician in the field of aging, both in research and practice. The award was established in 1977 through a bequest from a patient’s estate as a tribute to Dr. Joseph T. Freeman. The Excellence in Rehabilitation of Aging Persons Award is designed to acknowledge outstanding contributions in the field of the rehabilitation of aging individuals.

